# Neural control of fasting-induced torpor in mice

**DOI:** 10.1038/s41598-019-51841-2

**Published:** 2019-10-29

**Authors:** Timna Hitrec, Marco Luppi, Stefano Bastianini, Fabio Squarcio, Chiara Berteotti, Viviana Lo Martire, Davide Martelli, Alessandra Occhinegro, Domenico Tupone, Giovanna Zoccoli, Roberto Amici, Matteo Cerri

**Affiliations:** Department of Biomedical and Neuromotor Sciences, Alma Mater Studiorum - University of Bologna, Bologna, Italy

**Keywords:** Homeostasis, Neurophysiology, Brain, Neural circuits, Autonomic nervous system

## Abstract

Torpor is a peculiar mammalian behaviour, characterized by the active reduction of metabolic rate, followed by a drop in body temperature. To enter torpor, the activation of all thermogenic organs that could potentially defend body temperature must be prevented. Most of these organs, such as the brown adipose tissue, are controlled by the key thermoregulatory region of the Raphe Pallidus (RPa). Currently, it is not known which brain areas mediate the entrance into torpor. To identify these areas, the expression of the early gene c-Fos at torpor onset was assessed in different brain regions in mice injected with a retrograde tracer (Cholera Toxin subunit b, CTb) into the RPa region. The results show a network of hypothalamic neurons that are specifically activated at torpor onset and a direct torpor-specific projection from the Dorsomedial Hypothalamus to the RPa that could putatively mediate the suppression of thermogenesis during torpor.

## Introduction

Torpor is a peculiar mammalian behaviour, characterized by the active reduction of metabolic rate, with a consequent drop in body temperature proportional to the thermal gradient between the body and the environment^1^. Torpor is an ancestral trait, probably already present in proto-mammals^2^; it served as a strategy to survive in an environment with low resource availability^3^. Mice, being facultative heterotherms^4^, can display torpor when a negative energy balance is sensed^5^. In laboratory conditions, multiple strategies can be used to induce torpor in mice, mainly based on the reduction of ambient temperature and/or food deprivation/restriction^6–8^, but the neural pathway responsible for the entrance into torpor is unknown.

To enter torpor, all the thermogenic organs that could potentially mediate an attempt to compensate for the cooling of the body, such as the brown adipose tissue, must be turned off. Brown adipose tissue has been shown to be under the control of neurons located in the brainstem region of the Raphe Pallidus (RPa)^9^. RPa is a key relay in transmitting thermoregulatory commands from the central nervous system to the effectors, and, so far, no central signal has been reported to bypass the RPa relay station. This is a highly conserved region that has been shown to be functionally similar in many species, such as mice^10^, rats^11^, rabbits^12^, piglets^13^, and humans^14^. Since the suppression of thermogenesis is required in order to enter torpor, it is highly unlikely that RPa neurons can still be active in such a condition; an inhibitory afference to RPa neurons should therefore be activated at torpor onset.

To identify such an afference, the expression of the early gene c-Fos at torpor onset was assessed in different brain regions in animals in which a retrograde tracer (Cholera Toxin subunit b, CTb) had been previously injected into the RPa region. To obtain a clearly identifiable, reputable, and reliable torpor onset, we used a protocol described by Heldmaier and coworkers^7^. Briefly, mice subjected to a 36-h fasting period were acutely exposed to a cold environment. Animals entered torpor within two hours from the exposure.

Combining neurons expressing the early gene c-Fos with the expression of the retrograde tracer CTb injected within the RPa, we show here a network of hypothalamic neurons that are specifically activated at torpor onset and a direct torpor-specific projection to RPa originating in the Dorsomedial Hypothalamus (DMH) that could putatively mediate the suppression of thermogenesis of torpor itself.

## Results

### c-Fos expression

**Figure 1:**
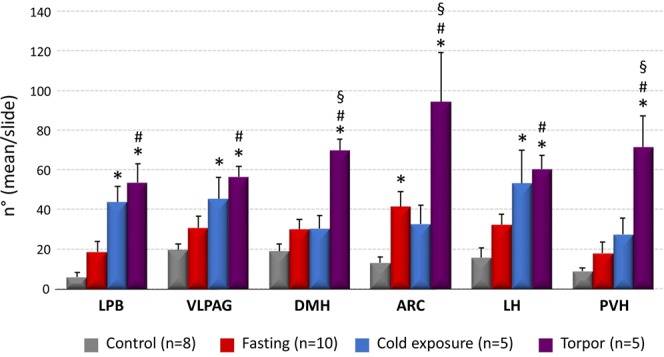
Average number (for slices of tissue) of c-Fos positive neurons in different brain regions. (LPB = Lateral Parabrachial Nucleus, VLPAG = Ventrolateral Periaqueductal Gray, DMH = Dorsomedial Hypothalamus, ARC = Arcuate Nucleus, LH = Lateral Hypothalamus, PVH = Paraventricular Nucleus of the Hypothalamus). *p < 0.05 vs. Control; ^§^p < 0.05 vs. Fasting group; ^#^p < 0.05 vs. all other conditions.

In the Torpor group, c-Fos was significantly more expressed compared with the other groups in the ARC (94.33 ± 24.97; P = 0.002 vs. Cold exposure, P = 0.003 vs. Fasting, P < 0.001 vs. Control), in the PVH (71.63 ± 15.40; P = 0.002 vs. Cold exposure, P < 0.001 vs. Fasting and vs. Control), and in DMH (69.83 ± 5.47; P < 0.001 for all comparisons) (Fig. 1). Within the ARC, there were significantly more c-Fos positive neurons (P = 0.048) in the Fasting group (41.46 ± 7.53) compared to the Control group (13.06 ± 2.83).

c-Fos expression within the LH, the LPB, and the VLPAG appears to be stimulated by cold exposure. In all these regions c-Fos positive cell count was higher in the Torpor group (LH: P = 0.02; LPB: P < 0.001; VLPAG: P = 0.01; vs. Fasting group) and in the Cold Exposure group (P < 0.001 vs. Control group for all comparisons). As expected, no c-Fos was detected in the RPa during torpor (Supplementary Fig. S1).

### c-Fos/CTb positive neurons

**Figure 2:**
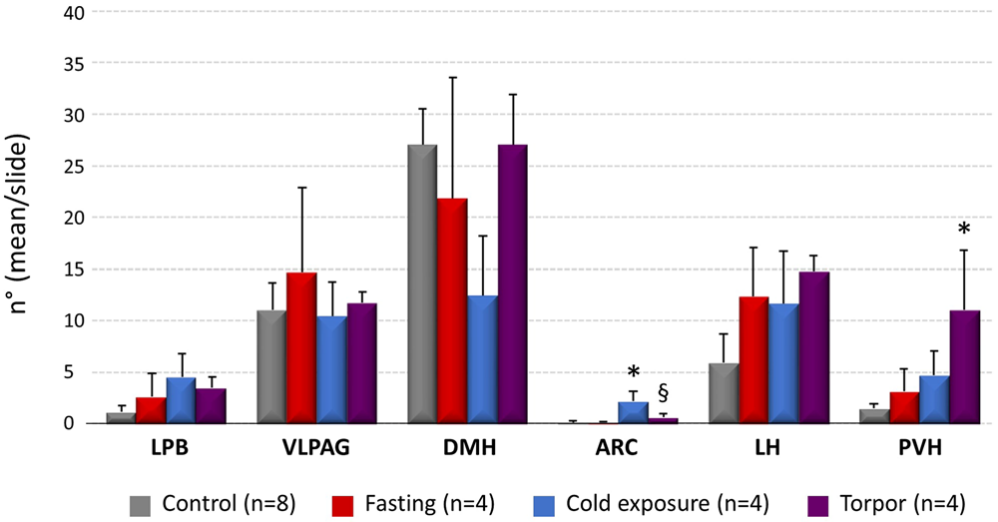
Average number (for slices of tissue) of CTb positive neurons in different brain regions. (LPB = Lateral Parabrachial Nucleus, VLPAG = Ventrolateral Periaqueductal Gray, DMH = Dorsomedial Hypothalamus, ARC = Arcuate Nucleus, LH = Lateral Hypothalamus, PVH = Paraventricular Nucleus of the Hypothalamus). *p < 0.05 vs. Control; ^§^p < 0.05 vs. Fasting group; ^#^p < 0.05 vs. all other conditions.

**Figure 3:**
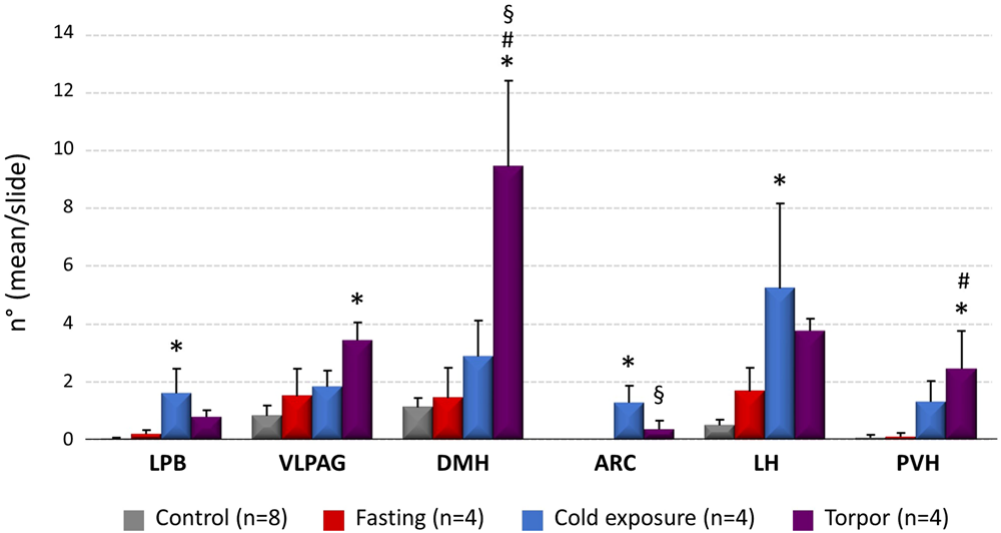
Average number (for slices of tissue) of CTb/c-Fos positive neurons in different brain regions. (LPB = Lateral Parabrachial Nucleus, VLPAG = Ventrolateral Periaqueductal Gray, DMH = Dorsomedial Hypothalamus, ARC = Arcuate Nucleus, LH = Lateral Hypothalamus, PVH = Paraventricular Nucleus of the Hypothalamus). *p < 0.05 vs. Control; ^§^p < 0.05 vs. Fasting group; ^#^p < 0.05 vs. all other conditions.

**Figure 4:**
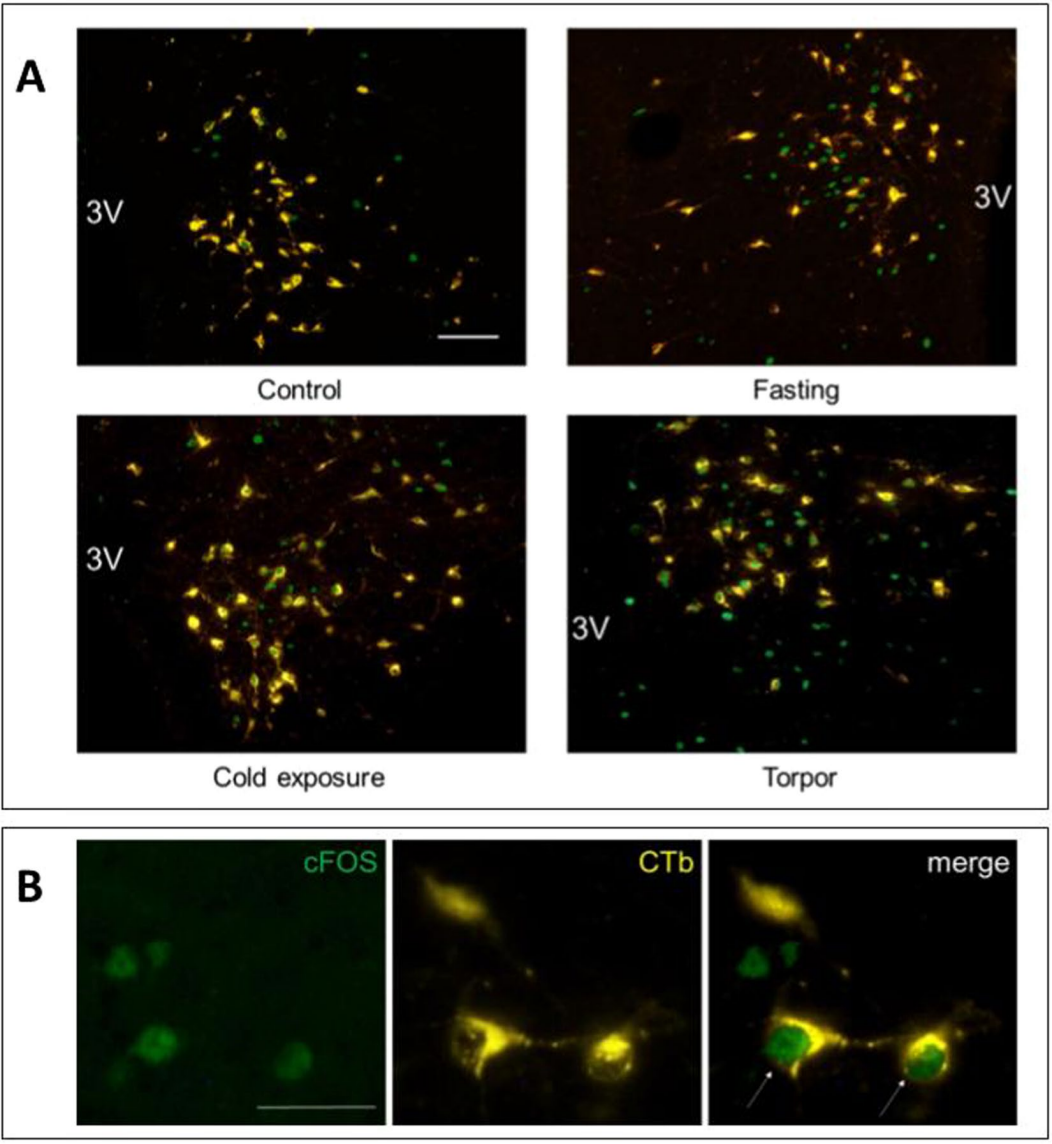
Panel A: exemplificative slices stained for c-Fos (green) and CTb (yellow) in the Dorsomedial Hypothalamus (DMH) in the four experimental groups; panel B: enlarged images of neurons stained for cFos (on the left), for CTb (in the center), and the merging of the previous two (on the right). 3 V = Third Ventricle. Horizontal bar = 50 µm.

No significant differences were detected in the distribution of CTb across the experimental groups (Fig. 2). In the Torpor group, the only region showing a significant increase in c-Fos positive neurons projecting to the RPa compared to the other groups was the DMH (9.45 ± 0.29; P = 0.012 vs. Cold exposure, P = 0.002 vs. Fasting, P < 0.001 vs. Control) (Figs 3 and 4).

**Figure 5:**
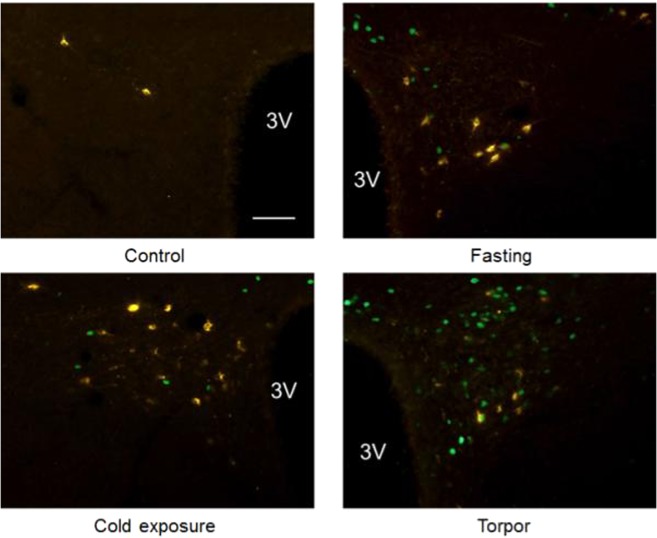
Exemplificative slices stained for c-Fos (green) and CTb (yellow) in the Paraventricular Nucleus of the Hypothalamus (PVH) in the four experimental groups. 3 V = Third Ventricle. Horizontal bar = 50 µm.

Within the PVH, significantly more RPa-projecting/c-Fos-positive neurons were present in the Torpor group (2.45 ± 0.12) compared to the Fasting group (0.1 ± 0.0; P = 0.025) and the Control group (0.07 ± 0.0; P = 0.012), but not in comparison with the Cold Exposure group (1.32 ± 0.2) (Figs 3 and 5). The VLPAG also showed a significant (P = 0.006), although modest, increase in torpor-related c-Fos/CTb positive neurons (3.42 ± 0.62) compared to the Control group (0.82 ± 0.36). No significant c-Fos/CTb positive neurons were observed in the ARC (Supplementary Fig. S2).

### Neurochemical characterization

In order to identify the neurochemical phenotype of the torpor-related neurons projecting to the RPa, we proceeded to perform the following stains in selected hypothalamic areas (Table 1):

1. MCH in LH. No triple-stained neurons were observed in the Lateral Hypothalamus (Supplementary Fig. S3).
2. Orexin in LH. Both in the Torpor group and in the Fasting group, orexin/c-Fos/CTb positive neurons were observed in the LH area, as were Orexin/c-Fos positive neurons. In the Cold Exposure group, only Orexin/c-Fos positive neurons were detected (Fig. 6).

**Figure 6:**
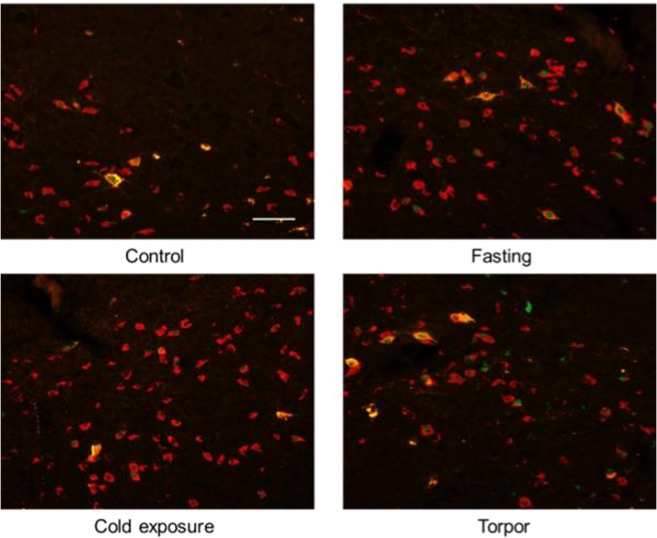
Exemplificative slices stained for c-Fos (green), CTb (yellow) and Orexin (red) in the Lateral Hypothalamus (LH) in the four experimental groups. Orx = Orexin. Horizontal bar = 50 µm.
3. ChAT in DMH. CTb was clearly expressed in neurons within the DMH, but in these neurons there was no colocalization with ChAT and c-Fos (Fig. 7).

**Figure 7:**
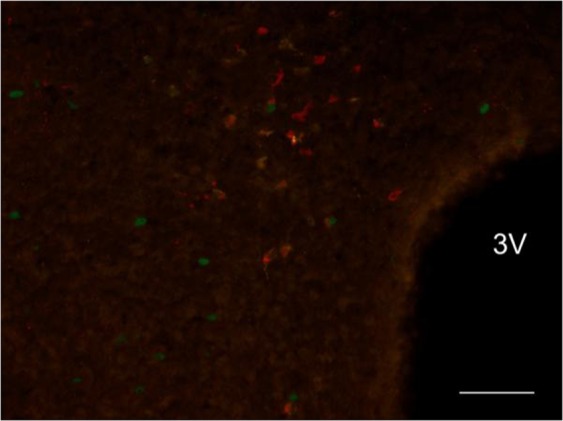
Exemplificative slice stained for c-Fos(green), CTb(yellow) and ChAT(red) in the Dorsomedial Hypothalamus (DMH) in the torpor group. 3 V = Third Ventricle; ChAT = Choline acetyltransferase. Horizontal bar = 50 µm.
4. TH in PVH. No neurons expressed TH and CTb or c-Fos (Supplementary Fig. S4).
5. POMC in ARC. POMC-expressing neurons were not positive for either CTb or c-Fos (Supplementary Fig. S5).
6. Oxytocin in PVH. No neurons expressed Oxytocin; neither were CTb or c-Fos expressed (Supplementary Fig. S6).

**Table 1:** Presence (+) or absence (−) of staining for Melanin Concentrating Hormone (MCH), Orexin, Choline acetyltransferase (ChAT), Tyrosine Hydroxylase (TH), Oxytocin, and Proopiomelanocortin (POMC) combined with c-Fos, Colerotoxin (CTb), or both in specific nuclei in all the experimental groups.

	MCH - LH			OREXIN -LH			ChAT - DMH			TH - PVH			OXYTOCIN - PVH			POMC - ARC		
	X-c-Fos	X-c-Fos-CTb	X-CTb	X-c-Fos	X-c-Fos-CTb	X-CTb	X-c-Fos	X-c-Fos-CTb	X-CTb	X-c-Fos	X-c-Fos-CTb	X-CTb	X-c-Fos	X-c-Fos-CTb	X-CTb	X-c-Fos	X-c-Fos-CTb	X-CTb
TORPOR	−	−	−	+	+	+	−	−	+	-	−	−	−	−	-	−	−	−
COLD-FED	−	−	−	+	−	+	−	−	+	−	−	−	−	−	−	−	−	−
WARM-FASTED	−	−	−	+	+	+	−	−	+	−	−	−	−	−	−	−	−	−
CONTR OL	−	−	−	−	−	+	−	−	+	−	−	−	−	−	−	−	−	−

(LH = Lateral Hypothalamus, DMH = Dorsomedial Hypothalamus, PVH = Paraventricular Nucleus of the Hypothalamus, ARC = Arcuate Nucleus).

## Discussion

The main result of this study is the identification of a neural network which is active at torpor onset in mice. This supports the hypothesis that torpor onset is neurally mediated, possibly through a direct modulation of the RPa, a key thermoregulatory area in the brainstem.

The protocol used to induce torpor combines two different stimuli, one tonic, fasting, and one phasic, cold exposure. The pattern of neuronal activation in these different conditions provides useful information regarding the processing of these two signals at the hypothalamic level, either independently or combined, in the induction of torpor.

The hypothalamic areas where the neurons that are selectively activated at torpor onset appear to be located are the ARC, the DMH, and the PVH. Among the activated neurons, the latter two regions also display a subgroup of neurons that directly project to the RPa.

The Arcuate nucleus is an area that is critically involved in food intake control^15^. Neurons in this area tonically express c-Fos during fasting, somehow in correlation with the sense of hunger^16^. From our data, in the Fasting group, there are, in fact, significantly more c-Fos positive neurons within the ARC compared to the Control group. It is, therefore, interesting that c-Fos positive neurons in the Torpor group are significantly higher in number than in the Fasting group. The addition of cold exposure to fasting in the Torpor group induced the activation of more ARC neurons on top of those activated by the tonic fasting only in the Fasting group; these neurons were apparently not induced by the cold exposure alone in the Cold exposure group, since the number of activated neurons in this group was not significantly higher than in the Control group. These neurons may be key to the induction of torpor. Considering that, within the ARC, fasting induces c-Fos expression in 25% of the NPY neurons^16^, and that NPY was shown to have a relevant role in modulating torpor^17,18^, it is possible to hypothesize that the extra ARC c-Fos neurons in the torpor group may well produce NPY, suggesting that torpor can be triggered only when a certain amount of NPY are activated.

At torpor onset, the Paraventricular nucleus also showed a large increase in c-Fos positive neurons that appear to be neither oxytocinergic nor THergic. In rats, the activation of neurons within the PVH was reported to inhibit the cold-induced activation of the brown adipose tissue^19^, supporting the hypothesis that neurons within this region can, directly or indirectly, inhibit thermogenesis and act as a node in the torpor-inducing network.

The Dorsomedial Hypothalamus is a key relay in transmitting thermoregulatory commands from hierarchically higher centres to the RPa^20^. In rats, activation of DMH neurons increases body temperature and heart rate^21^, but, in mice, a group of DMH neurons expressing ACh and projecting directly to the RPa was found to inhibit thermogenesis^22^. Even if, in anaesthetized rats, this pathway was shown to be tonically active^23^, it could be a possible candidate for a torpor-inducing pathway. DMH resulted as the only region showing a significantly higher number of c-Fos-positive neurons projecting to RPa in the torpor group compared to each of the three “control” groups; however, these neurons do not express ACh, ruling out the hypothesis that this pathway was mediating the entrance into torpor. Despite not being AChergic, we propose that this DMH-RPa projection is the pathway that is responsible for the inhibition of thermogenesis and entrance into torpor.

Acute cold exposure also activated several different areas, which do not appear to be specifically involved in torpor onset: the LPB, the VLPAG, and the LH.

The LPB was shown to directly process cold sensory inputs from the skin and to provide the hypothalamus with the resulting information^24^. Since, in our torpor model, cold exposure acted as a trigger, it is likely that LPB efference can, in a fasting animal, activate entrance into torpor instead of cold defence, similarly to what has been described as “thermoregulatory inversion” by Tupone and co-workers^25^.

The VLPAG has been shown to be anatomically and functionally connected with hypothalamic thermoregulatory centers^26–30^, but the role of this area in the control of body temperature is not wholly clear at the moment. It is interesting to note that within the VLPAG a small group of neurons is located that is active at torpor onset and projects to the RPa; these neurons may potentially be part of the torpor network.

The Lateral Hypothalamus is a variegated area involved in many physiological functions, including sleep^31^, food intake^32^, and thermoregulation^33^. Considering this area as a whole, the increase in the number of c-Fos positive neurons both in the Cold Exposure group and in the Torpor group suggests that the role of this area during torpor may somehow be linked to some residual thermoregulatory function. Some features of torpor could, indeed, be interpreted as the result of the activity of cold-activated ascending pathways. The EEG rhythm in torpor, for instance, differs from the traditional slow wave pattern typical of NREM sleep: prominent EEG frequencies slow down with the decreasing temperature^34^, possibly resembling the wake-like desynchronized pattern which characterizes wakefulness. Within the LH, the orexinergic neurons are involved in the promotion of wakefulness^35,36^, food intake^37^, and thermogenesis^38^. In rats, orexin microinjection within the RPa can further stimulate an already active thermogenic drive^38^, but, in our experiment, no c-Fos-positive, RPa projecting, orexinergic neurons were found in the Cold exposure group. Surprisingly, these triple-stained neurons were found both in the Fasting, and in the Torpor group. This suggests that the Orexin-RPa projecting neurons play a role that is more related to the modulation of hunger or food-seeking behavior^39^ than to thermoregulation. The brain area where orexin acts in order to stimulate food intake has not yet been identified, but the region of the RPa has been suggested as a possible candidate^40^.

In conclusion, several groups of neurons, mostly at a hypothalamic level, were identified as being specifically active at torpor-onset, providing the first detailed description of the network which may be responsible for the neuronal control of this state. The understanding of the characteristics of these neurons may pave the way for the identification of new possible pharmacological targets to mimic torpor, or to induce a state of synthetic torpor in non-hibernators^41^.

## Methods

### Ethical approval

All the experiments were conducted following approval by the National Health Authority (decree: No.141/2018 - PR/AEDB0.8.EXT.4), in accordance with the DL 26/2014 and the European Union Directive 2010/63/EU, and under the supervision of the Central Veterinary Service of the University of Bologna. All efforts were made to minimize the number of animals used and their pain and distress.

### Housing and surgery

Experiments were performed on 28 C57BL/6J wild-type female mice (it is well known that female mice are more susceptible to entering torpor than males) of 13–15 weeks old (The Jackson Laboratory), each weighing approximately 17–24 g. After their arrival, animals were housed for one week under standard laboratory conditions: light-dark cycle (LD cycle) 12 h:12 h (L 09:00–21:00) with ad libitum access to food (4RF21 diet, Mucedola, Settimo Milanese, Italy) and water, at an ambient temperature (Ta) of 25 ± 1 °C.

According to Oelkrug and colleagues (Oelkrug *et al*., 2011), when mice that are adapted to a neutral ambient temperature are deprived of food for 36 hours and then acutely exposed to a low ambient temperature, they display torpor shortly after (with longer bouts in dark phases). A similar protocol for torpor induction was observed here; after one week of adaptation to standard laboratory conditions, mice were moved to a different room, with the same ambient conditions as before, apart from the Ta (28 ± 1 °C) and the 12:12 LD cycle with lights on at 3 pm (i.e., Zeitgeber Time 0, ZT 0), that was shifted in order to create favorable conditions for inducing torpor at around ZT 20, corresponding to the 8th hour of the D period. Mice were kept under these laboratory conditions for two weeks. After this period of time they underwent surgery.

Mice were first anaesthetized with isoflurane 1–2% (Abbott S.p.a., Pomezia Roma, Italy, in pure oxygen) and injected with 200 μl of Rimadyl (Carprofen 5 mg/ml, Pfizer), and then positioned on a stereotaxic frame (Kopf instruments) not endowed with a gas delivery system; from that moment on, the anaesthetic was administered intraperitoneally (Ketamine, Imalgene 1000, Merial 100 mg/kg and xylazine, Xilor, Bio 98 Srl, 20 mg/kg). Once a good and stable electrocardiogram (ECG) signal was obtained via non-invasive electrodes, a glass micropipette, previously filled with N-methyl-d-aspartate (NMDA)(0.2 nM) was stereotaxically inserted in the RPa, −6.3 mm posterior to bregma, 0.0 mm lateral to the midline, −5.2 mm ventral to the dorsal surface of the cerebellum. The DV coordinate was then functionally tested by microinjecting NMDA. The approximate injected total volume was about 60 nl.

**Figure 8:**
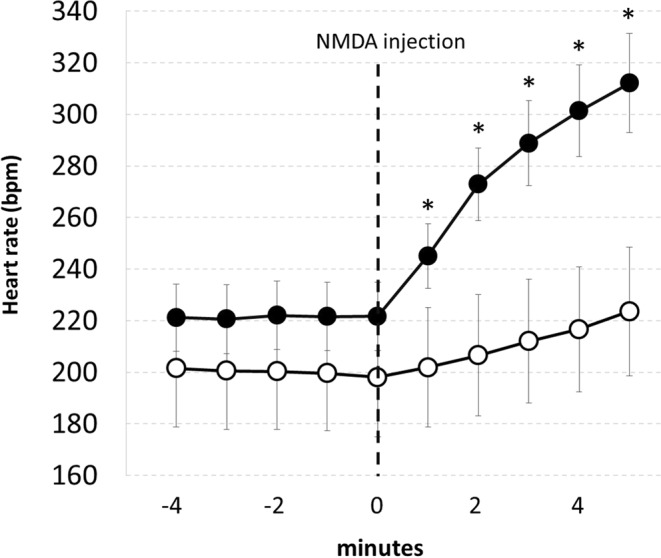
NMDA injection in the Raphe Pallidus. Average effect (mean ± SEM) induced by a microinjection of 60 nl of NMDA 0.2 nM within the Raphe Pallidus in anaesthetized mice. Solid circles referred to the group of animals with a correct location of the CTb tracer within the Raphe Pallidus (n = 20); Empty circles referred to animals with a suboptimal localization of the CTb tracer within the RPa (n = 8).

**Figure 9:**
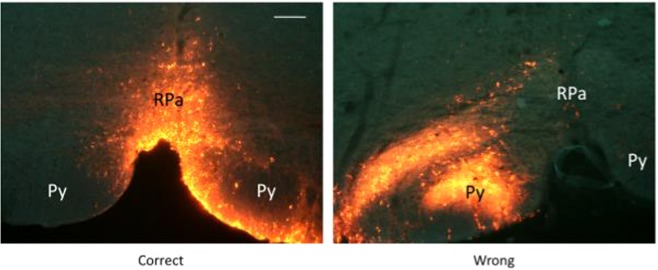
Cholera toxin B injection in the Raphe Pallidus. An example of a correct injection of the retrograde tracer Cholera toxin B subunit (CTb) within the Raphe Pallidus (RPa) (right), and of a discated injection (left). Py = Pyramids. Horizontal bar = 50 µm.

Since the NMDA delivered in the RPa should cause a consistent, rapid and reversible increase in heart rate^19^, the position of the micropipette was considered correct if this event occurred within two minutes from the end of the injection. In the eventuality of no physiological response to the drug, the micropipette was removed, and the coordinates were adjusted. We performed a maximum of three attempts for any one mouse. After obtaining a successful RPa localization, the pipette was removed and filled with a solution containing a monosynaptic retrograde tracer (Cholera Toxin b, CTb, 1 mg/ml), with fluorescent microbeads (Fluospheres carboxylate - modified, 0.1μm, red (580/605), Life Technologies, diluted 1:10, 2 μl in 50 μl of CTb) added in order to allow for post-mortem localization of the injection site. The micropipette was inserted again in the brain, and the second injection procedure was carried out as described above. In spite of a somehow positive functional response to the NMDA test, as described later in the immunohistochemistry section, in 8 animals the location of CTb was suboptimal. In Fig. 8 is shown the effect induced by NMDA injection in the RPA on HR in animals (n = 20) with a correct localization of CTb, and with a suboptimal localization of the injection (n = 8). An example of a correct and not correct CTb injection is shown in Fig. 9.

The skin surface was sutured, and mice were administered with broad spectrum antibiotic (30 μl of veterinary Rubrocillin, Intervet, Schering-Plough Animal Health, Milano; benzathine benzylpenicillin 12500 U.I./kg + dihydrostreptomycin sulphate 5 mg/kg: 140 U.I./mouse + 60 μg/mouse diluted in 800 μl of saline solution). The animals were kept under observation until the appearance of the first signs of recovery from general anaesthesia and then placed in their cage and back to the LD switched (ZT = 0 at 3 pm) and Ta = 28 °C, for a week, to recover from surgery. The animals’ pain, distress, or suffering symptoms were constantly monitored and evaluated using the Humane End Point (HEP) criteria.

### Torpor protocol

**Figure 10:**
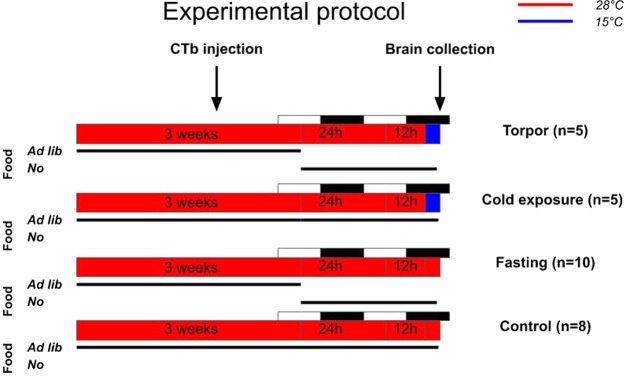
Protocol. Experimental protocol for the four experimental groups (Torpor, Cold exposure, Fasting, and Control).

Mice were divided into four experimental groups (Fig. 10):

(i) Torpor (n = 5): animals underwent fasting for 36 hours at Ta 28 °C, and, still with no access to food, on the experimental day (at ZT = 18.00, i.e. 9.00 am), acutely exposed to a lower Ta (15 °C), by moving their own cage to a different thermoregulated box, at Ta = 15 °C. The 36-h fasting combined with the acute exposure to a lower Ta (15 °C) caused the appearance of torpor, usually within 90–120 minutes (ZT = 19.30–20.00, approximately) (Fig. 4).
(ii) Cold exposure (n = 5): these mice were exposed for 36 h to Ta 28 °C and acutely exposed on the experimental day (at ZT = 18) to a lower Ta (15 °C). Food and water were always available *ad lib*. None of them displayed a torpid state.
(iii) Fasting (n = 10): These mice were deprived of food for 36 hours, and not exposed to a low Ta. None of them displayed a torpid state.
(iv) Control (n = 8): Mice were maintained at Ta 28 °C with food and water ad lib. for the entire duration of the experiment. None of them displayed a torpid state.

**Figure 11:**
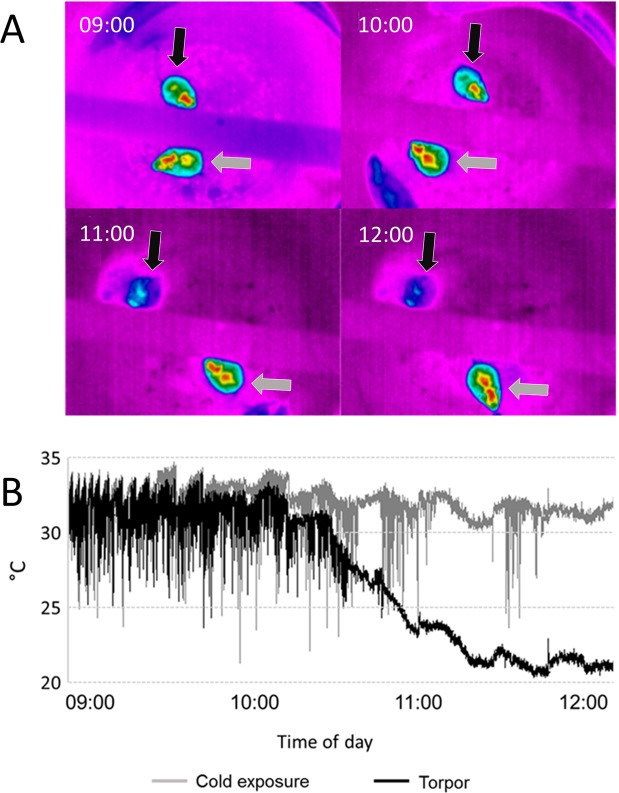
Cutaneous temperature recording during torpor. (**A**) Exemplificative thermographic images taken every hour from the exposure to the 15 °C environment. Top mouse, black arrow = Torpor group; bottom mouse, grey arrow = Cold exposure group. (**B**) An example of the recording of the maximum value of skin temperature during the experimental day, monitored by means of a thermal camera positioned above the cage. Changes induced by the exposure to a 15 °C environment (at 9:00) are shown in the Torpor group (black) and in the Cold exposure group (gray).

After confirming the presence of torpor (usually around 10.30–11.00 am) in the Torpor group, by visually observing the infrared images of the mouse recorded by an infrared thermal camera (Thermovision A20, Flir Systems) positioned above the experimental cage (Fig. 11), the condition was maintained and constantly monitored for 90 minutes. This time span was used because the expression of the early gene c-Fos reaches its peak within 90 minutes^42^. For the other three groups, the time frames were adjusted according to those of the Torpor group, to avoid any circadian discrepancy. Mice were then perfused (paraformaldehyde 4%) and brains were extracted for immunohistochemical analysis.

Since the rationale of the experiment is based on the idea that RPa neurons are inhibited during torpor, two non-operated mice were perfused after 1,5 and 6 hours in torpor that had been induced using the same protocol used for the Torpor group, in order to assess whether the RPa is active during torpor.

### Immunohistochemistry

In correspondence with the experimental conditions chosen, under general anesthesia mice were transcardially perfused with 50 ml of saline solution (NaCl 0,9%, w/v) followed by an equal amount of 4% (w/v) paraformaldehyde solution in sodium phosphate buffer (PBS). Brains were extracted and post-fixed for 2 hours by immersion in the same perfusion solution. Then samples were put in a 30% (w/v) sucrose solution in PBS and sodium-azide 0,02% (w/v) overnight for cryoprotection. Hereafter, tissue samples were embedded in a cryostat cutting medium (Killik) and cut into 35 µm-thick slices in a cryostat-microtome (Frigocut 2800), kept at −22,0 °C. All the slices were then stored, until analyzed, at −80 °C in a cryoprotectant solution: 30% (w/v) sucrose, 30% (v/v) ethylene glycol, 1% (w/v) polyvinylpyrrolidone in PBS.

The following neuronal structures were analyzed, considered from caudal to rostral direction: Lateral Parabrachial Nucleus (LPB); Ventrolateral part of the Periaqueductal Gray matter (VLPAG); Lateral Hypothalamus (LH); Arcuate Nucleus of the Hypothalamus (Arc); Dorsomedial Nucleus of the Hypothalamus (DMH); Paraventricular Nucleus of the Hypothalamus (PVH).

Two steps of immunohistochemistry were carried out: i) firstly, staining c-Fos expression together with CTb to find the neuronal nuclei that were activated and that, potentially, projected to the RPa; ii) with a special focus on the specifically-activated structures found in the previous step, activated neurons were phenotypically studied.

For both steps, a sample of 1 out of 6 slices of the whole brain were used for immunostaining. Every slice set was rinsed twice in PBS and then incubated at room temperature for 2 hours in 1% (v/v) normal donkey serum blocking solution.

### First step – c-Fos/CTb

Of the 28 animals used, 8 showed a suboptimal localization of the CTb and were excluded from this part of the study, therefore number of animal for each group changed to: Torpor n = 4, Fasting n = 4, Cold Exposure n = 4, Control n = 8. Following the blocking solution, all slices were incubated at room temperature overnight with the following primary antibodies: i) goat Anti-CTb (List Biological Laboratories); ii) rabbit Anti-c-Fos (Calbiochem). Both primary antibodies were diluted at 1:10000. Slices were then rinsed twice in PBS with 0,3% (v/v) Triton X-100 and incubated at room temperature for 2 hours with the following secondary antibodies: i) Donkey Anti-goat IgG conjugated with Alexa-555 (Thermo Fisher); ii) Donkey Anti-rabbit IgG conjugated with Alexa-488 (Thermo Fisher). Both secondary antibodies were diluted at 1:500. Finally, tissue slices were mounted on coated glass slices and coverslipped with an anti-fade mounting medium (ProLong Gold mountant; Thermo Fisher).

### Second step – neuronal typization

For this step, considering that some primary antibodies were from the same host, after the first blocking solution triple-staining was carried out as follows:

1. Slices were incubated overnight at room temperature with the following primary antibodies: for Arc, rabbit Anti-pro-opiomelanocortin (POMC; Phoenix Pharmaceuticals; 1:500); for LH, rabbit Anti-melanin concentrating hormone (MCH; Phoenix Pharmaceuticals; 1:5000) or rabbit Anti-orexin A (Orx; Calbiochem; 1:5000); for PVH, monoclonal rabbit Anti-oxytocin (Oxt; abcam; 1:1000).
2. Slices were then rinsed twice in PBS with 0,3% (v/v) Triton X-100 and incubated for 2 hours with Donkey Anti-rabbit IgG conjugated with Alexa-594 (Thermo Fisher; 1:500).
3. Slices were then rinsed twice in PBS with 0,3% (v/v) Triton X-100 and incubated for 2 hours in 3% (v/v) normal rabbit serum.
4. Slices were then rinsed twice in PBS with 0,3% (v/v) Triton X-100 and incubated overnight at room temperature with donkey Anti-rabbit monovalent Fab fragment (Jackson ImmunoResearch Lab; 1:50).
5. Slices were then rinsed twice in PBS with 0,3% (v/v) Triton X-100 and incubated overnight at room temperature with: i) goat Anti-CTb (List Biological Laboratories; 1:10000); ii) rabbit Anti-c-Fos (Calbiochem; 1:10000).
6. Slices were then rinsed twice in PBS with 0,3% (v/v) Triton X-100 and incubated for 2 hours at room temperature with Donkey Anti-rabbit IgG conjugated with Alexa-488 (Thermo Fisher; 1:500) and Donkey Anti-goat IgG conjugated with Alexa-555 (Thermo Fisher; 1:500).
7. Finally, tissue slices were mounted on coated glass slices and coverslipped with an anti-fade mounting medium (ProLong Gold mountant; Thermo Fisher).

This staining protocol was also carried out on the DMH, but with a goat Anti-choline acetyltransferase (ChAT; Merk-Millipore) as the first primary antibody and a normal goat serum 3% (v/v) for the saturation step (i.e., the previous point 3).

For the PVH, as the first primary antibody a mouse-Anti tyrosine hydroxylase (TH; Immunostar) was used. Since, in this case, there was no host overlapping, no saturation steps (i.e., the previous points 3 and 4) were carried out. In any case, in order to limit possible interactions among the different primary antibodies, the procedure followed the two steps described above: Anti-TH – Alexa-594 – Anti-c-Fos/Anti-CTb – Alexa 488/555.

### Image acquisition and analysis

Images were obtained with a Nikon eclipse 80i equipped with Nikon Digital Sight DS-Vi1 color camera, at 200x magnification. Each microscopic field of interest was acquired in separate pictures, one per fluorochrome used. Multiple-staining pictures were obtained off-line by merging the different single-stained pictures.

Positive immunoreactions and multiple staining were counted using ImagePro Analyzer 7.0 (Media Cybernatics). Considering the 1 out of 6 slices taken for each animal, all the brain slices presenting the neuronal structures of interest were counted and averaged. Therefore, for each structure, every animal provided a mean value per slice, that was averaged with the same values obtained from the other animals belonging to the same experimental condition.

### Statistical analysis

Data were analyzed separately for each brain structure considered. Statistical analysis was carried out only on cFos and CTb data, using a one-way ANOVA (SPSS 25.0), considering the experimental conditions as the between-groups parameter. To compare means of the different experimental groups a modified t-test (t*)^43^. was used, considering the following comparisons: Torpor group vs. all the other experimental conditions, Control vs. Cold Exposure group and Control vs. Fasting group. Significance level was pre-set at p < 0.05.

The datasets generated and/or analysed during the current study are available from the corresponding author upon reasonable request.

## Supplementary information

Supplementary figures
