# Author Correction: Neural control of fasting-induced torpor in mice

**DOI:** 10.1038/s41598-020-61223-8

**Published:** 2020-03-03

**Authors:** Timna Hitrec, Marco Luppi, Stefano Bastianini, Fabio Squarcio, Chiara Berteotti, Viviana Lo Martire, Davide Martelli, Alessandra Occhinegro, Domenico Tupone, Giovanna Zoccoli, Roberto Amici, Matteo Cerri

**Affiliations:** Department of Biomedical and Neuromotor Sciences, Alma Mater Studiorum - University of Bologna, Bologna, Italy

Correction to: *Scientific Reports* 10.1038/s41598-019-51841-2, published online 29 October 2019

In this Article, there is a repeated typographical error in the legends of Figure 1, 2 and 3 where,

“^§^p  <  0.05 vs. Fasting group; ^#^p  <  0.05 vs. all other conditions.”

should read:

“^#^p  <  0.05 vs. Fasting group; ^§^p  <  0.05 vs. all other conditions.”

