# Supplementary material for: Neural control of fasting-induced torpor in mice
Source: Sci Rep. 2019 Oct 29;9:15462. doi: 10.1038/s41598-019-51841-2 (PMC6820542; doi:10.1038/s41598-019-51841-2)
Supplement: Supplementary file 1 — Supplementary figures [file 41598_2019_51841_MOESM1_ESM.docx]

**Neural control of fasting-induced torpor in mice.**

*Timna Hitrec, Marco Luppi, Stefano Bastianini, Fabio Squarcio, Chiara Berteotti, Viviana Lo Martire, Davide Martelli, Alessandra Occhinegro, Domenico Tupone, Giovanna Zoccoli, Roberto Amici, Matteo Cerri**

Timna Hitrec - Department of Biomedical and Neuromotor Sciences, Alma Mater Studiorum - University of Bologna. timna.hitrec@gmail.com

Marco Luppi - Department of Biomedical and Neuromotor Sciences, Alma Mater Studiorum - University of Bologna. marco.luppi@unibo.it

Stefano Bastianini - Department of Biomedical and Neuromotor Sciences, Alma Mater Studiorum - University of Bologna. stefano.bastianini3@unibo.it

Fabio Squarcio - Department of Biomedical and Neuromotor Sciences, Alma Mater Studiorum - University of Bologna. [fabio.squarcio2@unibo.it](mailto:fabio.squarcio2@unibo.it)

Chiara Berteotti - Department of Biomedical and Neuromotor Sciences, Alma Mater Studiorum - University of Bologna. chiara.berteotti@unibo.it

Viviana Lo Martire - Department of Biomedical and Neuromotor Sciences, Alma Mater Studiorum - University of Bologna. viviana.lomartire2@unibo.it

Davide Martelli - Department of Biomedical and Neuromotor Sciences, Alma Mater Studiorum - University of Bologna. d.martelli@unibo.it

Alessandra Occhinegro - Department of Biomedical and Neuromotor Sciences, Alma Mater Studiorum - University of Bologna alessandr.occhinegr2@unibo.it.

Domenico Tupone - Department of Biomedical and Neuromotor Sciences, Alma Mater Studiorum - University of Bologna. domenico.tupone@gmail.com

Giovanna Zoccoli - Department of Biomedical and Neuromotor Sciences, Alma Mater Studiorum - University of Bologna. giovanna.zoccoli@unibo.it

Roberto Amici - Department of Biomedical and Neuromotor Sciences, Alma Mater Studiorum - University of Bologna. roberto.amici@unibo.it

* Matteo Cerri - Department of Biomedical and Neuromotor Sciences, Alma Mater Studiorum - University of Bologna. matteo.cerri@unibo.it

**Supplementary figures**

**c-Fos in the Raphe Pallidus**

***
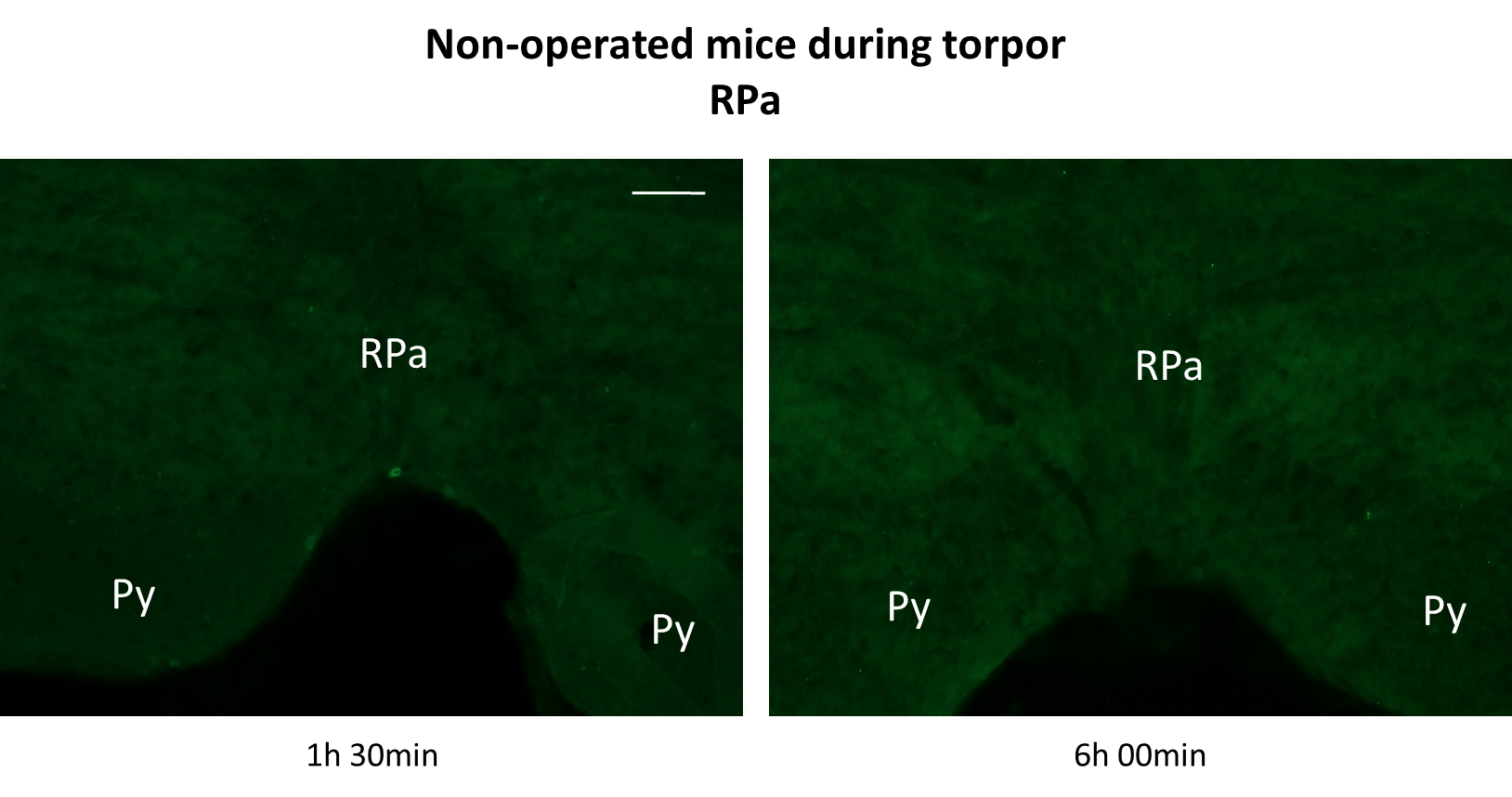
***

**Supplementary Figure S1.** Example of c-Fos staining (green) in the area of the Raphe Pallidus (RPa) 90 minutes after torpor (right panel) and 360 minutes after torpor (left panel). Py = Pyramids. Horizontal bar = 50µm

CTb cFos
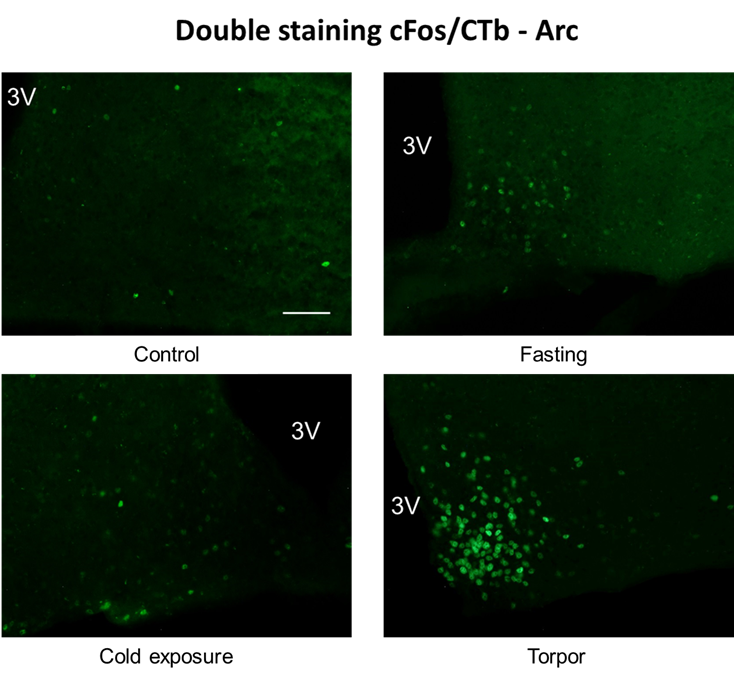


**Supplementary Figure S2.** Exemplificative slices stained for c-Fos (green) and CTb (yellow) in the Arcuate Nucleus (Arc) in the four experimental groups. 3V = Third Ventricle. Horizontal bar = 50µm

**Lateral Hypothalamus panoramic vision**

**
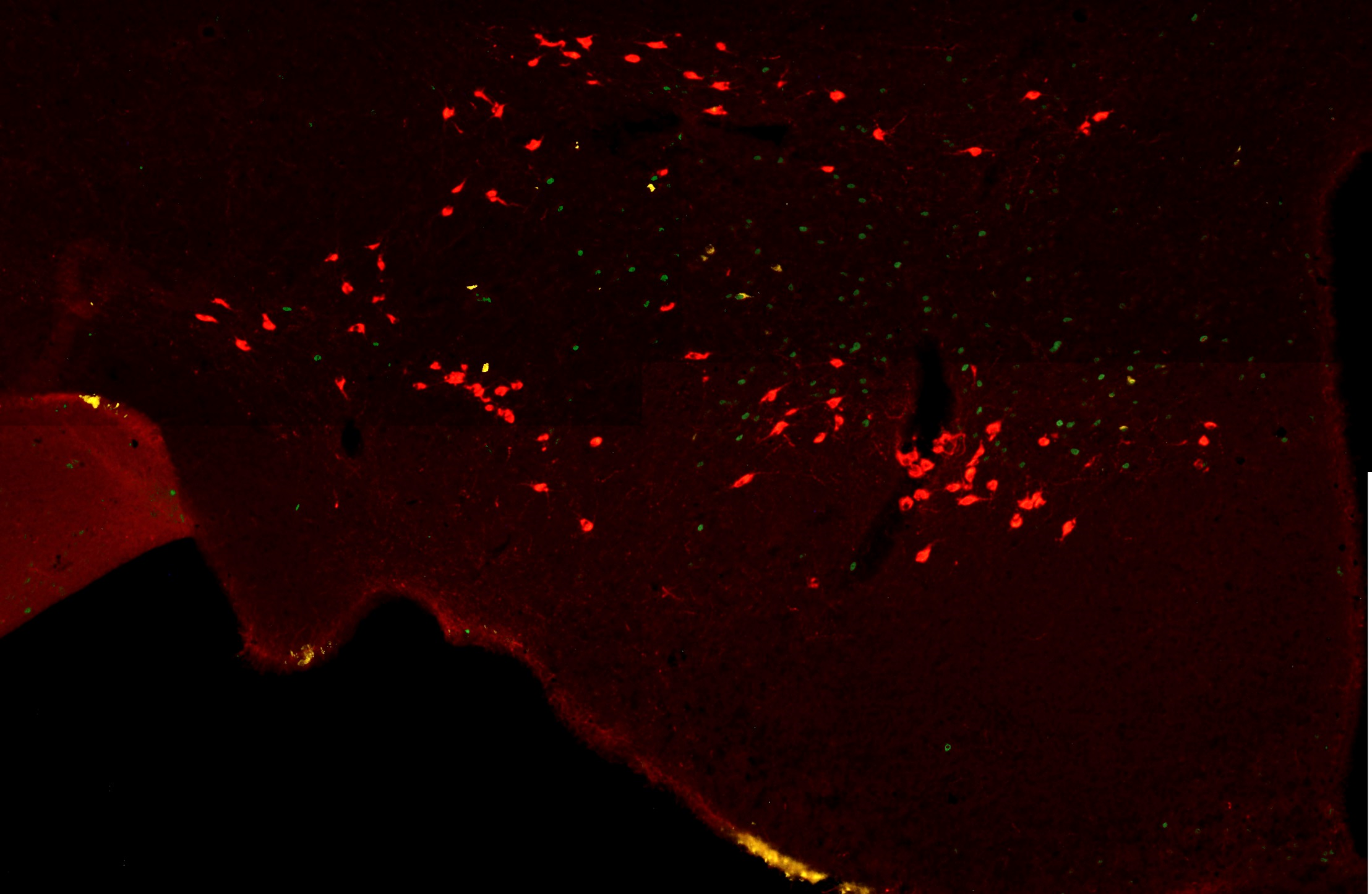
**

MCH CTb cFos

**Supplementary Figure S3.** Panoramic view of the Lateral Hypothalamus stained for Melanin Concentrating Hormone (red), Cholera-toxin b (CTb)(yellow) and c-Fos (green) in the Torpor group

**Paraventricular Nucleus panoramic vision**


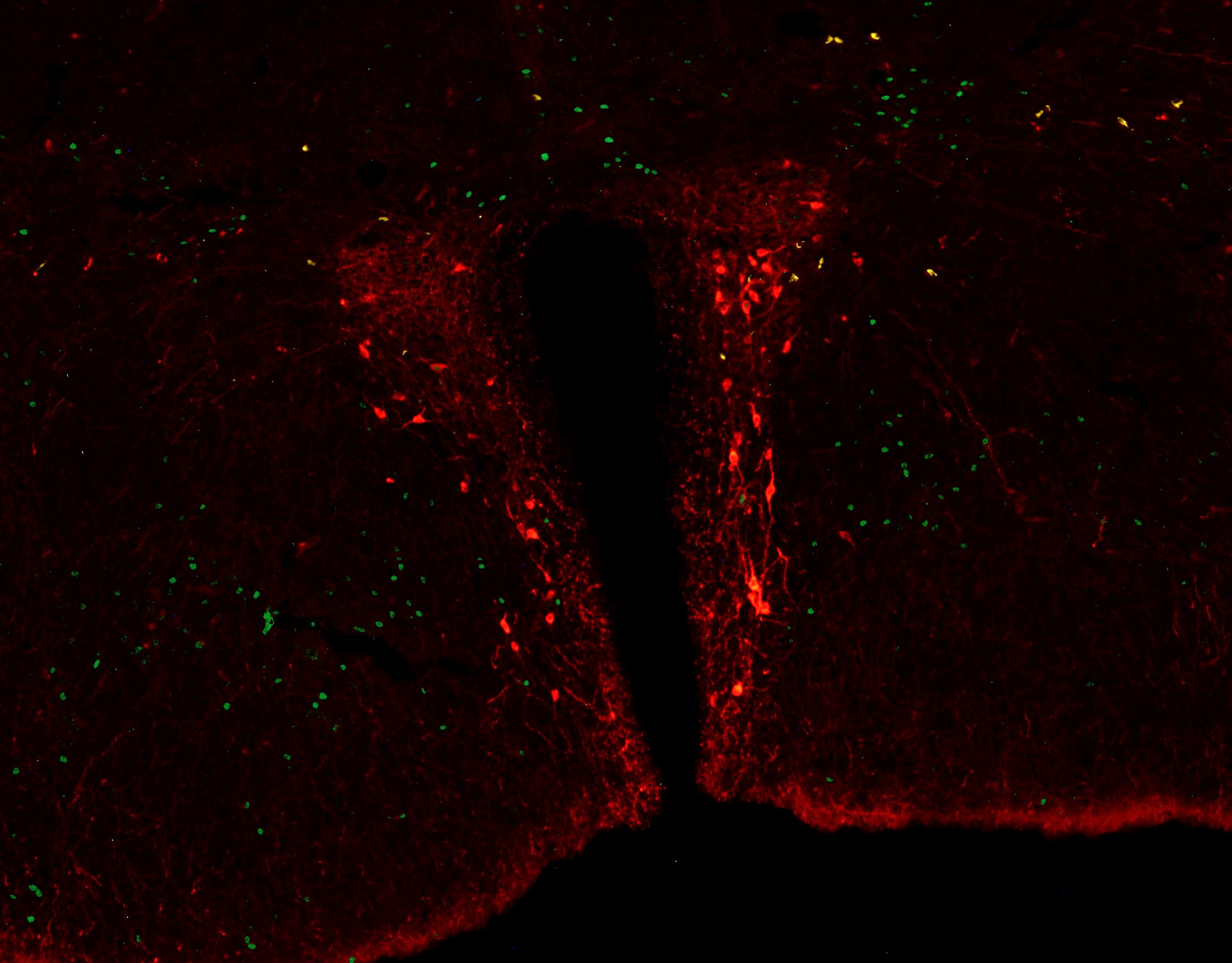


TH CTb cFos

**Supplementary Figure S4.** Panoramic view of the Paraventricular Nucleus of the Hypothalamus stained for Tyrosine Hydroxylase (red), Cholera-toxin b (CTb)(yellow) and c-Fos (green) in the Torpor group.


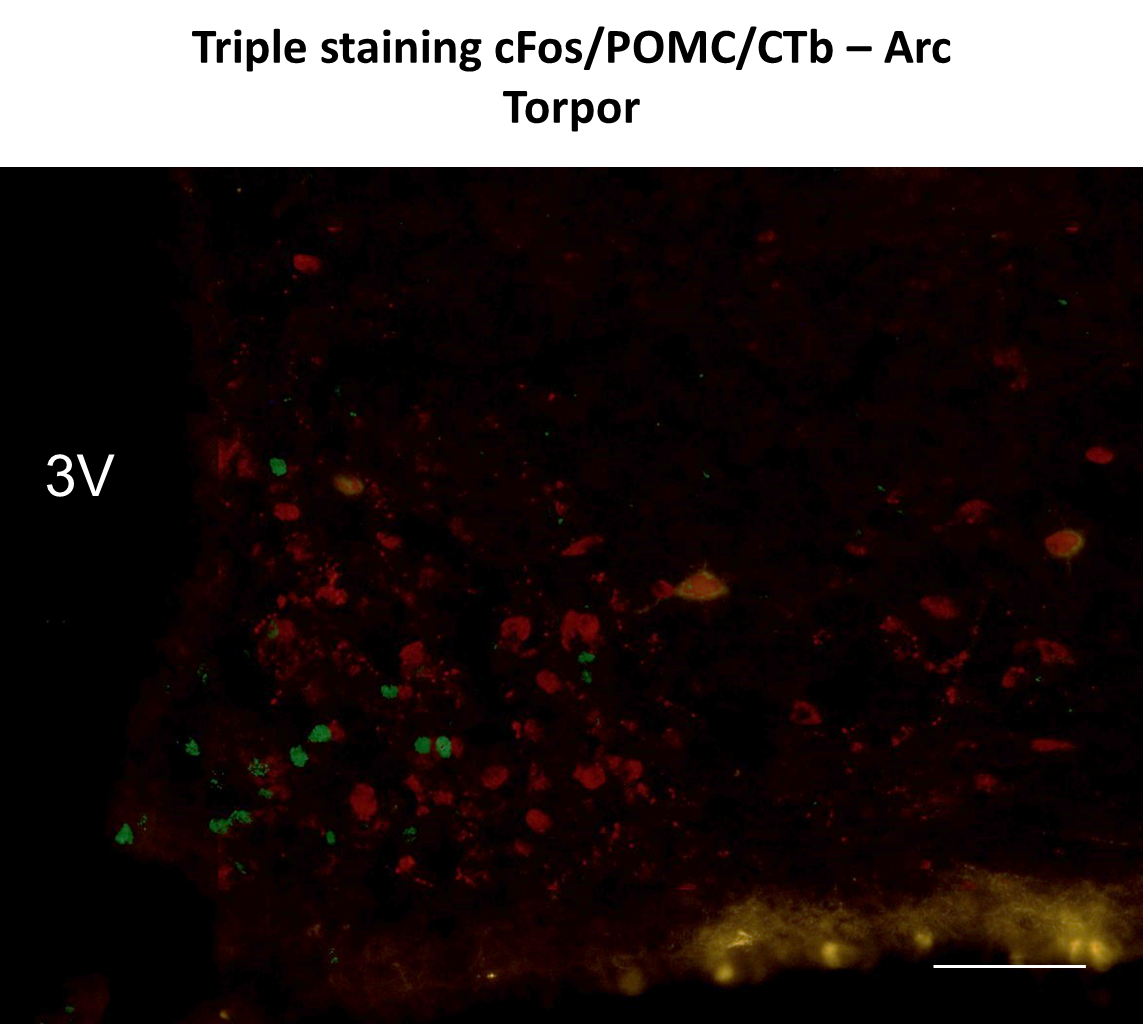


POMC CTb cFos

**Supplementary Figure S5.** Exemplificative slice stained for c-Fos (green), CTb(yellow) and POMC(red) in the Arcuate Nucleus (Arc) in the torpor group. 3V = Third Ventricle; POMC = Proopiomelanocortin. Horizontal bar = 50µm

**Paraventricular Nucleus panoramic vision**


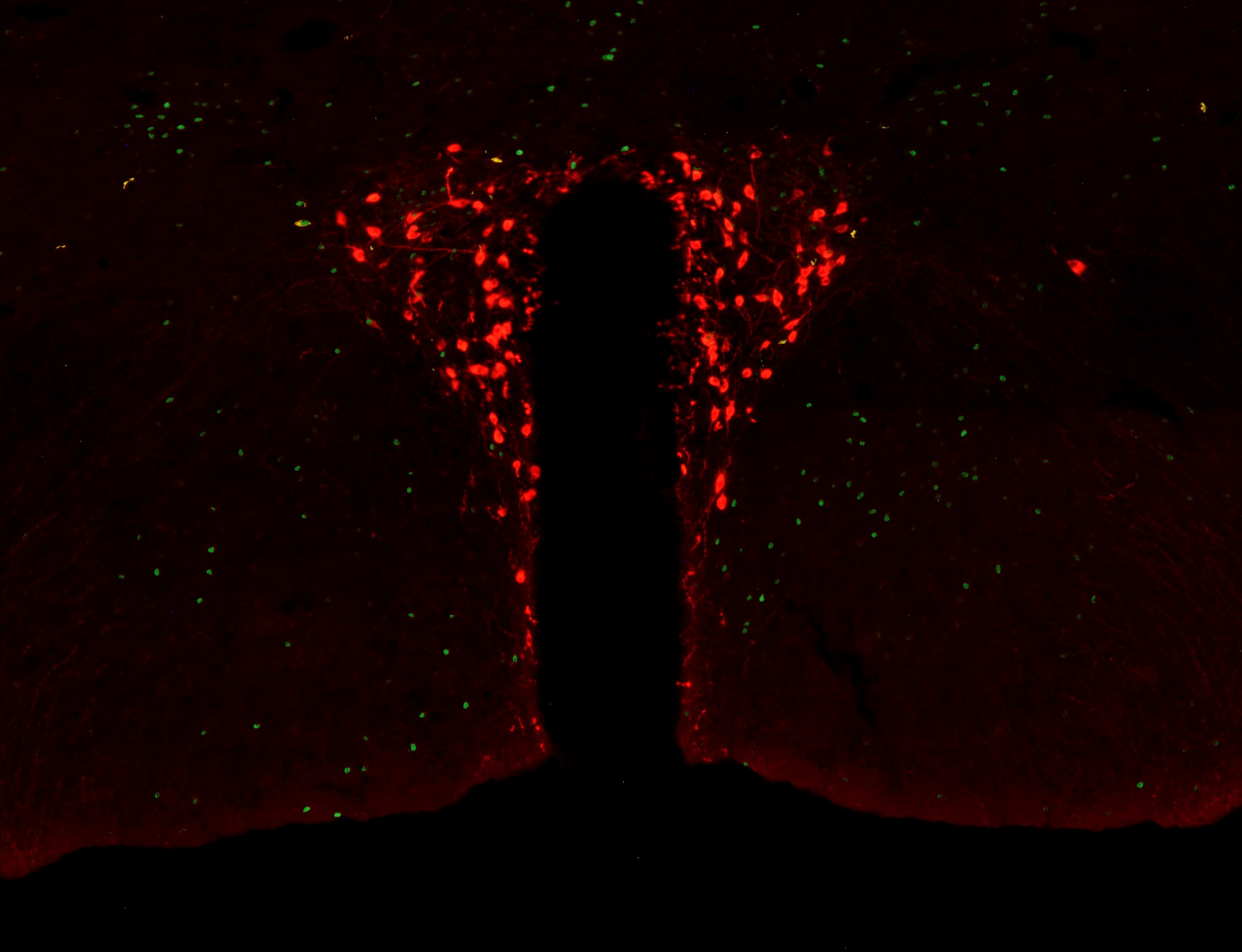


Oxy CTb cFos

**Supplementary Figure S6.** Panoramic view of the Paraventricular Nucleus of the Hypothalamus stained for Oxytocin (red), Cholera-toxin b (CTb)(yellow) and c-Fos (green) in the Torpor group.
